# Development of a time-resolved mirrorless scintillation detector

**DOI:** 10.1371/journal.pone.0246742

**Published:** 2021-02-12

**Authors:** Wonjoong Cheon, Hyunuk Jung, Moonhee Lee, Jinhyeop Lee, Sung Jin Kim, Sungkoo Cho, Youngyih Han

**Affiliations:** 1 Department of Health Sciences and Technology, SAIHST, Sungkyunkwan University, Seoul, Korea; 2 Proton Therapy Center, National Cancer Center, Goyang, Korea; 3 Department of Radiation Oncology, Virginia Commonwealth University, Richmond, Virginia, United States of America; 4 Department of Radiation Oncology, Samsung Medical Center, Seoul, Korea; 5 Department of Radiation Oncology, Samsung Medical Center, Sungkyunkwan University School of Medicine, Seoul, Korea; Dartmouth College Geisel School of Medicine, UNITED STATES

## Abstract

**Purpose:**

We developed a compact and lightweight time-resolved mirrorless scintillation detector (TRMLSD) employing image processing techniques and a convolutional neural network (CNN) for high-resolution two-dimensional (2D) dosimetry.

**Methods:**

The TRMLSD comprises a camera and an inorganic scintillator plate without a mirror. The camera was installed at a certain angle from the horizontal plane to collect scintillation from the scintillator plate. The geometric distortion due to the absence of a mirror and camera lens was corrected using a projective transform. Variations in brightness due to the distance between the image sensor and each point on the scintillator plate and the inhomogeneity of the material constituting the scintillator were corrected using a 20.0 × 20.0 cm^2^ radiation field. Hot pixels were removed using a frame-based noise-reduction technique. Finally, a CNN-based 2D dose distribution deconvolution model was applied to compensate for the dose error in the penumbra region and a lack of backscatter. The linearity, reproducibility, dose rate dependency, and dose profile were tested for a 6 MV X-ray beam to verify dosimeter characteristics. Gamma analysis was performed for two simple and 10 clinical intensity-modulated radiation therapy (IMRT) plans.

**Results:**

The dose linearity with brightness ranging from 0.0 cGy to 200.0 cGy was 0.9998 (R-squared value), and the root-mean-square error value was 1.010. For five consecutive measurements, the reproducibility was within 3% error, and the dose rate dependency was within 1%. The depth dose distribution and lateral dose profile coincided with the ionization chamber data with a 1% mean error. In 2D dosimetry for IMRT plans, the mean gamma passing rates with a 3%/3 mm gamma criterion for the two simple and ten clinical IMRT plans were 96.77% and 95.75%, respectively.

**Conclusion:**

The verified accuracy and time-resolved characteristics of the dosimeter may be useful for the quality assurance of machines and patient-specific quality assurance for clinical step-and-shoot IMRT plans.

## Introduction

Two-dimensional (2D) dosimeters have been studied for many years. Radiochromic films are used as absorbed dose dosimeters, and the most recent type, that is the Gafchromic EBT3 film (Ashland ISP Advanced Materials, NJ, USA), is widely applied clinically. The advantages of the EBT3 film are its high resolution and discoloration via a self-development process [1,2]. For accurate dosimetry, the EBT3 film must be managed extremely carefully [3–5], including its in-batch variation, film uniformity, and scanner dependency. For accurate dose measurement, various background subtraction methods have been proposed [6,7]. The EBT3 film is a single-measurement dosimeter; thus, multiple measurements require multiple films, and real-time dosimetry cannot be performed.

To perform 2D dosimetry while maintaining the advantages of the ionization chamber as a reference dosimeter, 2D ionization chamber arrays, such as MatriXX (Ion Beam Application, Belgium) and 2D-ARRAY seven29 (Physikalisch-Technische Werkstätten, Freiburg, Germany), have been developed [8–13]. The real-time 2D dose distribution can be obtained as an absolute dose through proper calibration; however, the spatial resolution is relatively coarse. In the case of MatriXX, the spatial resolution is 7.62 mm (center-to-center), and a resolution of 3.8 mm can be obtained by taking a second measurement after shifting the array position [14].

Since a pioneering report by S. N. Boon *et al*. published in 1998, research on 2D dosimetry using scintillator sheets has been actively conducted with X-ray and charged-particle dosimetry [15]. Scintillation detectors developed using inorganic 2D scintillators, organic 2D scintillators, and gas 2D scintillators have been extensively studied because such detectors have a high spatial resolution, reusability, and potential for time-resolved dosimetry because of their capability for real-time signal processing [16–18]. Conventional scintillation detectors usually consist of a scintillator, mirror, and scintillation acquisition device, such as a charge-coupled device (CCD) and complementary metal-oxide-semiconductor (CMOS) image sensors.

The mirror located in conventional scintillation detectors not only reflects the scintillation to the image sensor but also protects the camera from the primary beam. However, installing a mirror at 45° requires a strong and heavy holder to sustain the mirror; thus, the detector becomes heavy and bulky [15,19–30]. The underside of the scintillator should be empty because the light has to reach the image sensor. However, this causes a dose error ranging from 0.5 to 2.5% due to the lack of backscatter in 6 MV photon beams [31].

If a mirror is not installed in a scintillation detector system with an image sensor, several problems occur, including geometric distortions, brightness dependency, lack of backscatter, and penumbra distortion.

In this study, to build a compact 2D dosimeter, we removed the mirror and developed a time-resolved mirrorless scintillation detector (TRMLSD). Various issues caused by the removal of the mirror were corrected using image processing techniques for the correction of the geometric brightness dependency and by using a convolutional neural network (CNN) for backscatter and penumbra correction. It was observed that the TRMLSD could provide a high-precision 2D dose distribution in a plane perpendicular to the beam axis at 24 frames per second (fps).

## Materials and methods

### Design

The conventional scintillation detector comprises a scintillator, a mirror, optical fibers, and a camera with an image sensor. If an optical fiber is used to collect scintillation, the collected scintillation is not influenced by the scintillation from the environment. However, a 2D array structure requires a large number of optical fibers with a relatively low spatial resolution because of the thickness and array spacing of each optical fiber. When a mirror is used to reflect scintillation, the scintillation detector becomes larger and heavier.

**Fig 1:**
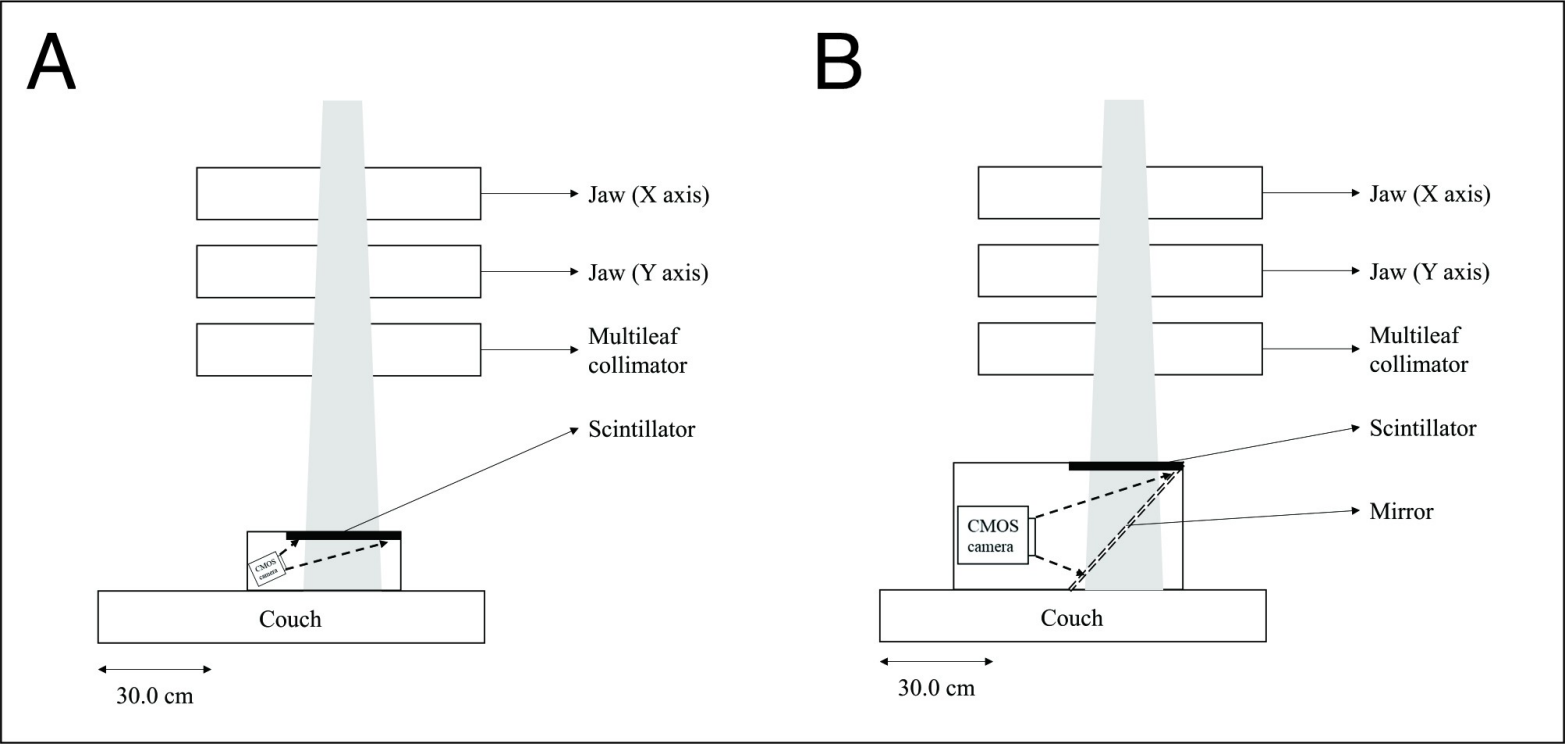
Diagram of the scintillation detector: (A) Developed TRMLSD and (B) conventional scintillation detector with a mirror.

To overcome these shortcomings, optical fibers and mirrors were removed. A camera with a CMOS image sensor was located outside the primary radiation field to protect it from damage due to radiation, and the camera was installed facing upwards at a certain angle. The detector and camera were held in place with a frame fabricated from Foamex plastic. The TRMLSD had a volume of 40.5 × 32.5 × 15.5 cm^3^, and a weight of 1.74 kg (Fig 1A), which was much smaller and lighter than the conventional type of scintillation detector developed in-house (Fig 1B, 60.7 × 37.0 × 33.5 cm^3^ and 18.26 kg). All the inner surfaces of the TRMLSD were covered with a dark film to prevent the reflection of light so that the camera could detect only the scintillation generated on the inorganic scintillator plate.

Nevertheless, scintillation detectors produce dosimetry errors owing to their structure. The scintillation generated from the scintillator installed on the TRMLSD must be recorded by the camera; therefore, the space under the scintillator is filled with air. This structural characteristic disrupts the electron equilibrium on the scintillator. Hu and Zhu *et al*. published a research paper related to the backscatter correction factor for megavoltage photon beams [31]. In that study, the dose loss at a depth of 5.0 cm (source–axis distance, SAD: 100.0 cm) in a water phantom was approximately 2.25% for a 6 MV photon beam. Moreover, we calculated the effect of backscatter using a Monte Carlo simulation toolkit [32]. The energy of the medical linear accelerator (linac, Novalis Tx; Varian Medical Systems, CA, USA) was 6 MV, the field size was 10.0 × 10.0 cm^2^, the standard source-to-surface distance (SSD) was 100.0 cm, and the water depth was 5.0 cm. The dose loss was approximately 2.5% due to the lack of electron backscatter.

#### Scintillator: PI-200

A flat-plate-type PI-200 inorganic 300 × 300 × 0.9 mm^3^ scintillator (Mitsubishi Chemical, Chiyoda, Japan) was selected because of its higher density and yield ratio than an organic scintillator. The main peak spectrum of the emitted scintillation was approximately 530 nm. The PI-200 scintillator layer was composed of three layers: protective, phosphor, and supporting layers.

Two problems exist when measuring the 2D dose distribution using an inorganic scintillator: (1) The PI-200 is a high-Z-material (gadolinium oxide sulfide doped with terbium) scintillator. The dose distribution obtained using the PI-200 is different from that obtained using a scintillator composed of a low-Z material, such as normal tissue and water. (2) Because the homogeneity of the chemical components constituting the three layers is not uniform, the uniformity of scintillation needs to be corrected using a homogeneous light source such as a high-quality large liquid crystal display (LCD) diagnostic monitor and/or multiple exposures with a large standard beam [29].

#### Scintillation measuring apparatus: GoPro HERO5 black

To measure the scintillation, a GoPro HERO5 Black (GoPro, CA, USA) camera was used. It is lightweight (118 g) and transmits signals wirelessly, enabling remote on/off control of the signal transmission. The scintillation distribution was converted into an image using the CMOS image sensor in the camera. Therefore, determining the camera parameters that are suitable for accurate measurement of the absorbed dose is important. In particular, the camera parameters related to exposure must be precisely selected; thus, the ISO value was carefully determined through iterative experiments to achieve reproducibility in dosimetry.

Conventionally, the ISO value represents the sensitivity of a photographic film to light. However, digital cameras also provide ISO settings. Depending on the ISO value, the sensitivity to light and the relative brightness vary. Therefore, based on the setting of the ISO value, the output factor of a linac measured by the TRMLSD varies. For a 5.0-cm-thick solid water phantom, the brightness differs by a factor of 1.99 between ISO values of 800 and 3200.

**Fig 2:**
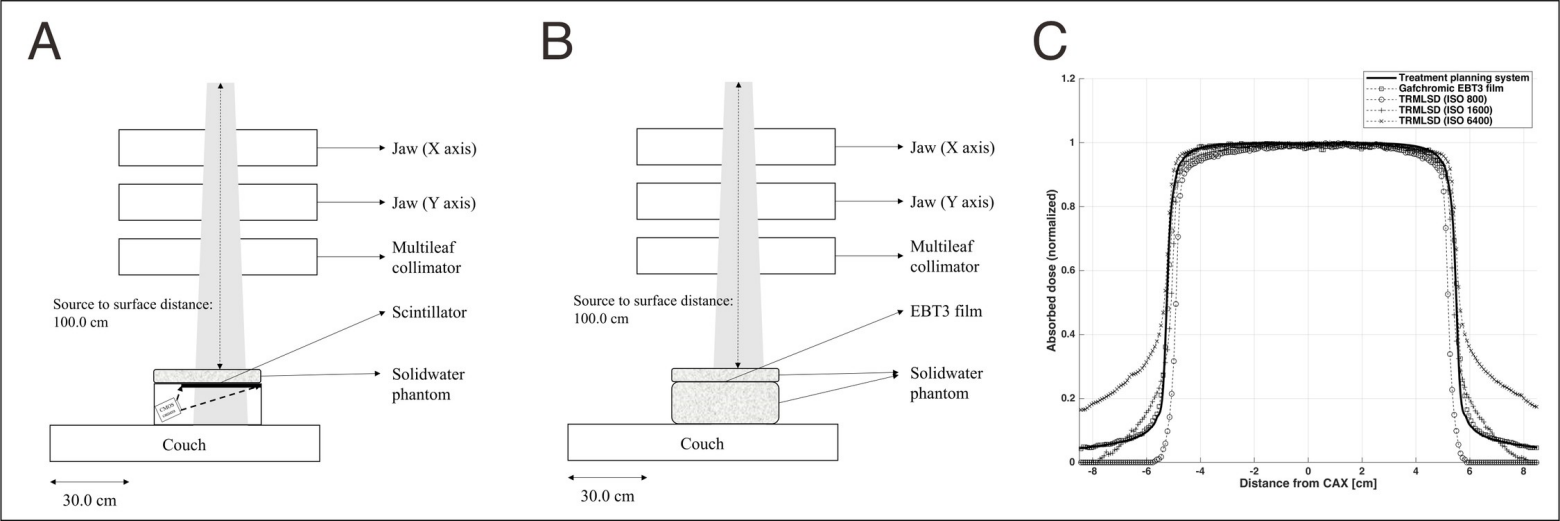
Experimental Setup for (A) the TRMLSD and (B) EBT3 film. (C) The measured profiles normalized to the maximum pixel value. Radiations were delivered at 249 monitor units with a 10.0 × 10.0 cm^2^ field for three different ISO values (800, 1600, and 6400) for the TRMLSD.

An ISO value of 800 was finally selected because it provided accurate output factors for the radiation field size in the iterative experiments. The experimental setup for the TRMLSD and EBT3 film was as follows: 249 monitor units (MUs) were delivered with a 10.0 × 10.0 cm^2^ field size to the solid water phantom at a distance of 100.0 cm from the source (SSD was 100.0 cm), and the scintillator or EBT3 film was positioned at a depth of 5.0 cm in the phantom. In Fig 2, Setup-A (Fig 2A) represents the scintillator in the TRMLSD setup, and Setup-B (Fig 2B) denotes the EBT3 film setup. Fig 2C depicts the lateral profiles normalized to the maximum pixel value of each measurement, which changes with the ISO values for the TRMLSD. The profile presented in Fig 2C was calculated using a treatment planning system for Setup-B (TPS, Pinnacle^TM^; Philips Medical Systems, CA, USA). The HERO5 Black provides the maximum ISO value of 6400; however, with this value, the dose in the outfield is greater than that of the EBT3 film measurements; therefore, ISO values of 800 and 1600 were tested. Other camera parameters, such as color, white balance, shutter speed, and exposure value, should be kept constant to ensure the reproducibility of the dose measurement. The image resolution in the video-recording mode was determined to be 4K resolution (3840 × 2160 pixels).

### Dosimetry from video

Scintillations were recorded in video mode at 24 fps with 4K resolution using the CMOS camera; then, the recorded data were divided into frames. In the case of the step-and-shoot IMRT plan, the beam was only turned on when the leaves of the multileaf collimator were stationary in each of the prescribed segment/subfield positions. Therefore, a time interval existed between the segments. To acquire the 2D dose distribution for each segment and/or field, all frames were reviewed, and the image frames with scintillation signals were grouped into intervals. During preprocessing, the recorded color images were converted into grayscale images. The major wavelength of the scintillator was approximately 530 nm; thus, the recorded image appeared green. The recorded pixel intensity ratios were approximately 10.5%, 51.5%, and 38.0% for the red, green, and blue channels, respectively. The weights of each color channel for the mix were fixed at a ratio of 1:1:1 to conserve the intensity of each color channel.

**Fig 3:**
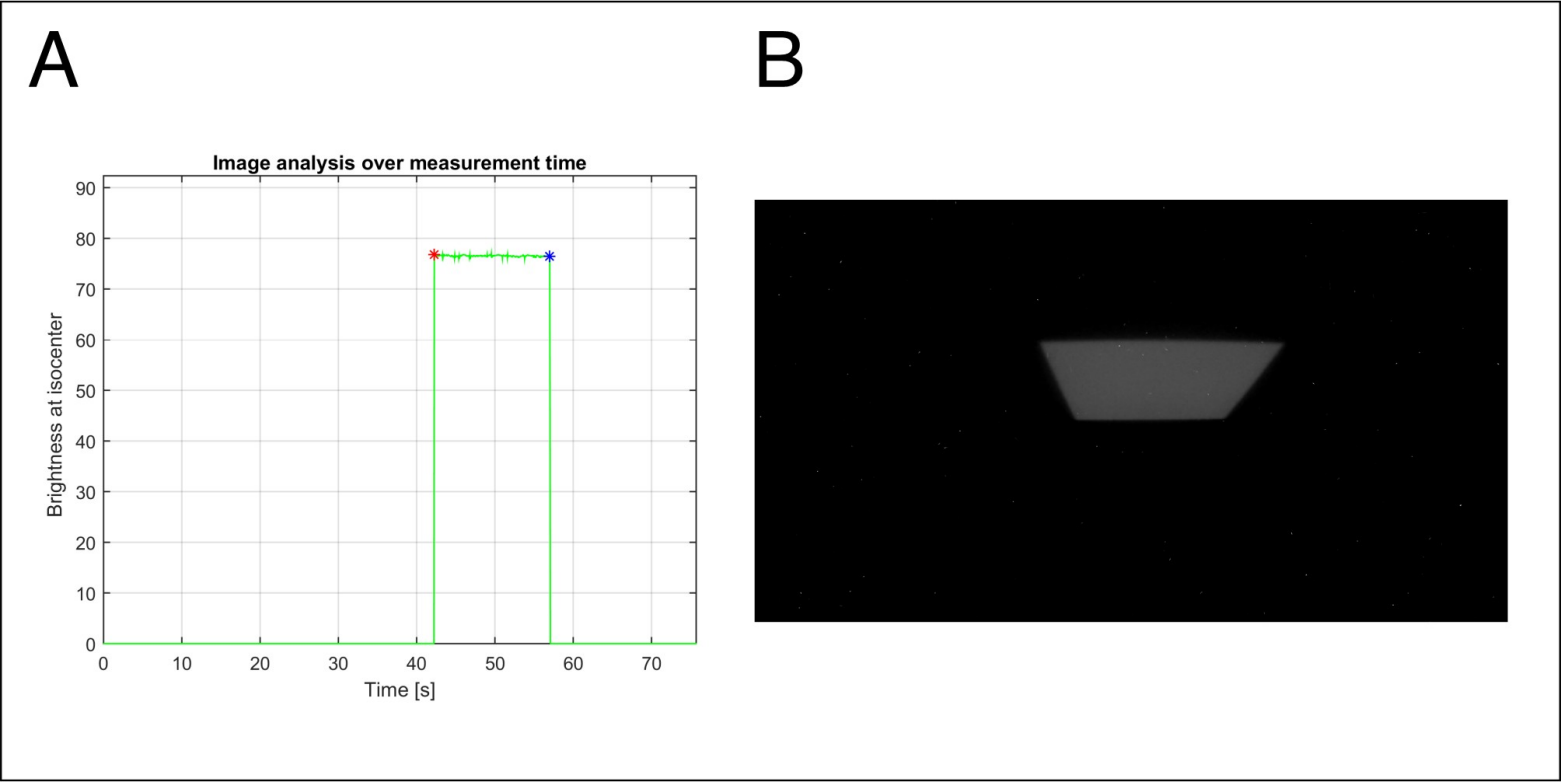
Analysis of the recorded scintillation distribution: (A) Signal of scintillation over time, recorded by the developed system. (B) Two-dimensional scintillation distribution captured by the HERO5 Black at 50.41 s.

To test the dosimetry capability, a 100 MU of a 6 MV X-ray beam with a 10.0 × 10.0 cm^2^ field was irradiated to a 5.0-cm-thick solid water phantom on top of the TRMLSD (Setup-A). Of the total video-recording period of 75.33 s (Fig 3A), the scintillation image was recorded for 14.88 s. Fig 3B shows the scintillation image taken at 50.41 s.

### Frame-based noise-reduction technique

**Fig 4:**
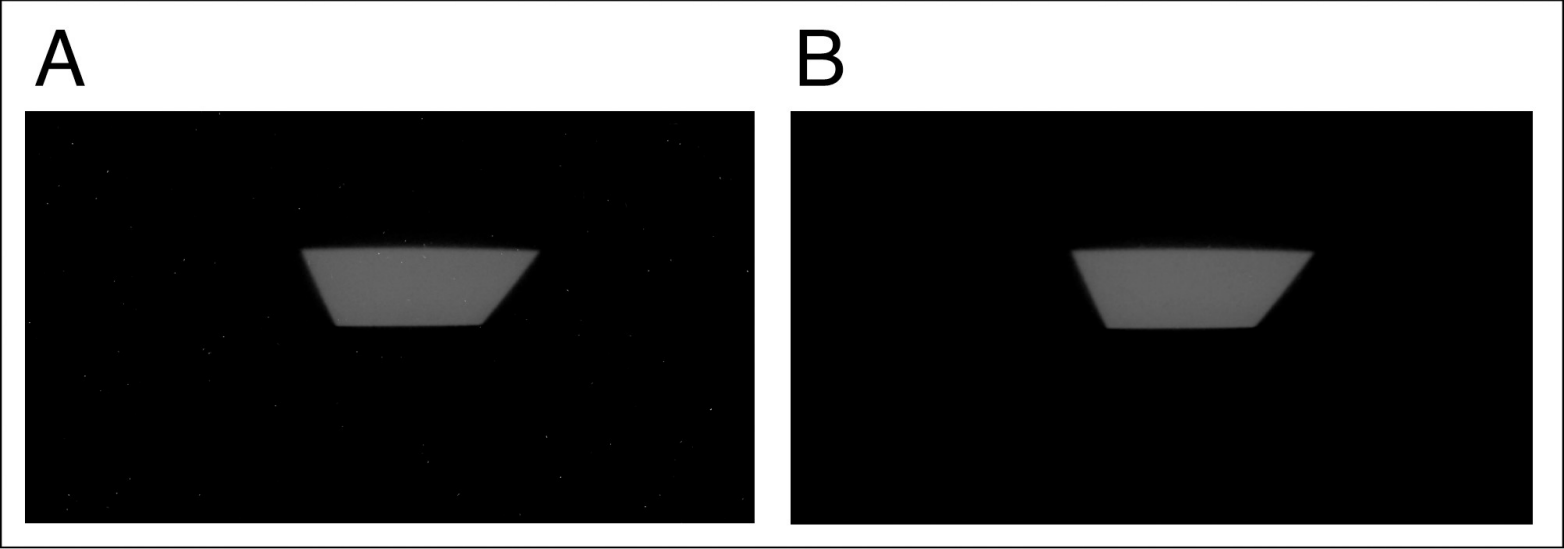
FBNR technique: (A) Original two-dimensional (2D) scintillation distribution, (B) 2D scintillation distribution after application of the FBNR technique.

Collimator- or phantom-scattered photons interact with the CMOS image sensor during measurement and generate hot pixels in the image, which are instantaneous, high-intensity random noise. The hot pixels were removed using instantaneous characteristics. The frame-based noise-reduction (FBNR) technique monitors all frames over time. It can detect and remove hot pixels by reading each pixel’s value from consecutive frames (Eq 1). If the value of pixel (*x*, *y*) in a certain frame is continuously present in several subsequent frames, the pixel is treated as a scintillation; otherwise, the pixel is treated as a hot pixel. The FBNR technique removes the hot pixels while maintaining the spatial resolution of the image without blurring, thus compensating for the drawbacks of noise-reduction techniques that use conventional filters, such as the median filter [19] or moving average filter [33]. The FBNR technique applied to the image is illustrated in Fig 4.

$$If\;\sum_{vfn=n}^{n+k}\;D(vfn,x,y)\geq Threshold\;value,D(vfn,x,y)≔0,$$ (1)

where *D* is the dose distribution measured by TRMLSD, *vfn* is the video frame number of interest for removing hot pixels, *(x*, *y)* are the pixel coordinates in the image, *n* is the scan starting point, and *k* is the number of frames for scanning. The *k* and threshold values, set to 5 and 3, respectively, were empirically determined by considering the computation time.

### Geometric correction for two-dimensional dose distribution

**Fig 5:**
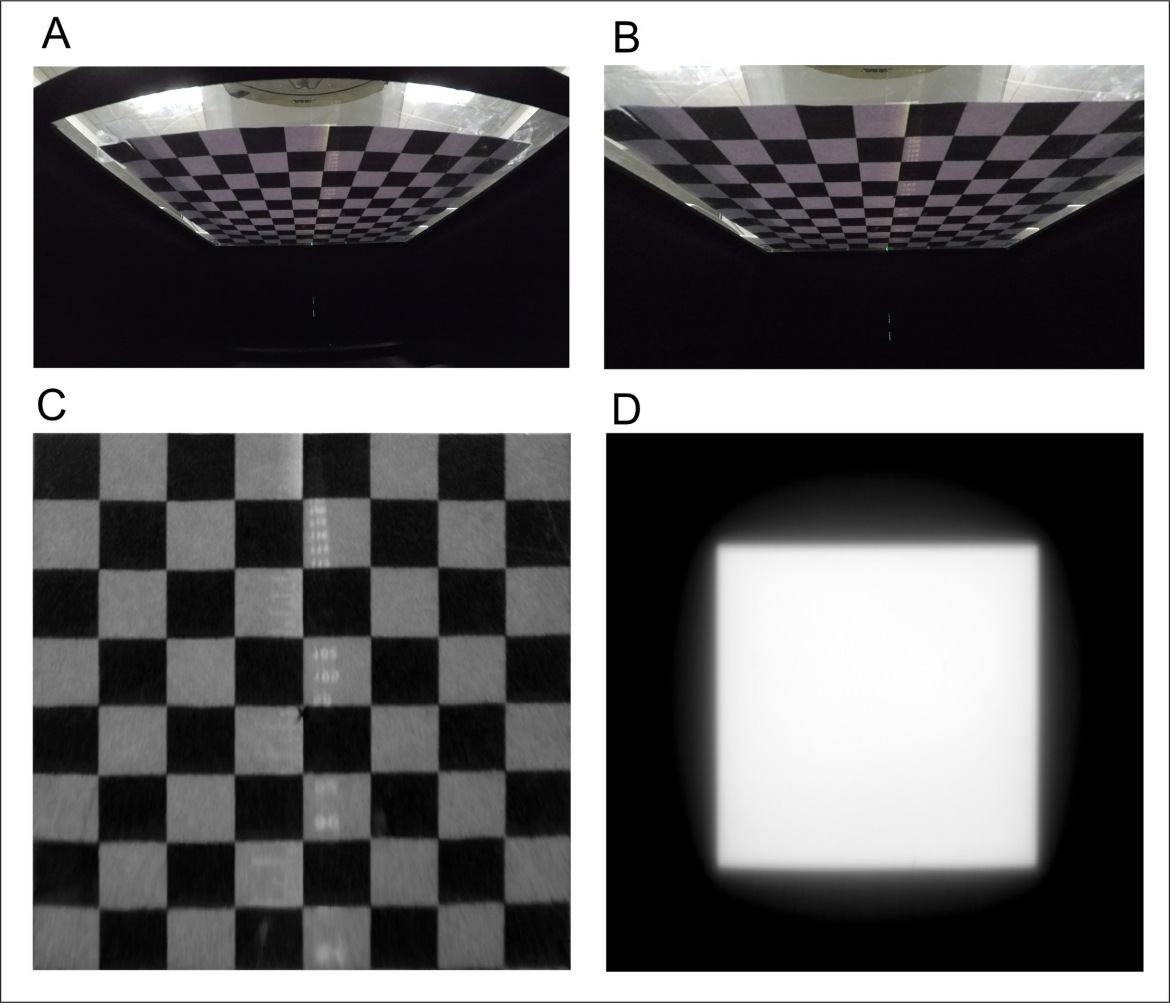
Procedure for geometric correction: (A) Original image, (B) image after lens correction, (C) image after perspective correction, and (D) two-dimensional scintillation distribution of the 10.0 × 10.0 cm^2^ field after geometric correction.

The 2D scintillation images obtained by the TRMLSD with the camera included the lens distortion caused by the curvature of the lens and the geometric perspective distortion generated by the angle of the camera lens with respect to the scintillator plate. The 2D dose distribution perpendicular to the beam axis was measured by correcting these two types of geometric distortions with correction algorithms utilizing a chessboard image. A chessboard pattern with squares of area 22.0 × 22.0 mm^2^ was placed at the position of the scintillator. After capturing an image of the chessboard pattern (Fig 5A), first, the lens distortion was corrected (Fig 5B), and then the perspective distortion was corrected using the ideal image coordinates of the chessboard (Fig 5C). Perspective correction transformed the tilted or skewed image into an image that would appear if the scintillation incidence was orthogonal to the camera. The geometric calibration had to be performed only once, and the correction parameters were stored as a geometry calibration file.

The geometric correction procedure was applied to the images obtained with 100 MU of 6 MV X-ray irradiation with a 10.0 × 10.0 cm^2^ field on a 5.0-cm-thick solid water phantom placed on the TRMLSD (Setup-A). The geometry-corrected radiation field of 17.6 × 17.6 cm^2^ had a resolution of 2048 × 2048 pixels, which corresponded to a spatial resolution of 0.086 mm (Fig 5D).

### Verification of relative brightness variation

The pyramid test pattern consisted of 2.0 × 2.0, 5.0 × 5.0, 10.0 × 10.0, and 20.0 × 20.0 cm^2^ fields with 100 MU for each field. The pyramid dose pattern was irradiated to assess the brightness variations because of the absence of a mirror, uniformity of the scintillator, and radiation field size. A solid water phantom with a thickness of 5.0 and 10.0 cm invariance was placed on the TRMLSD.

**Fig 6:**
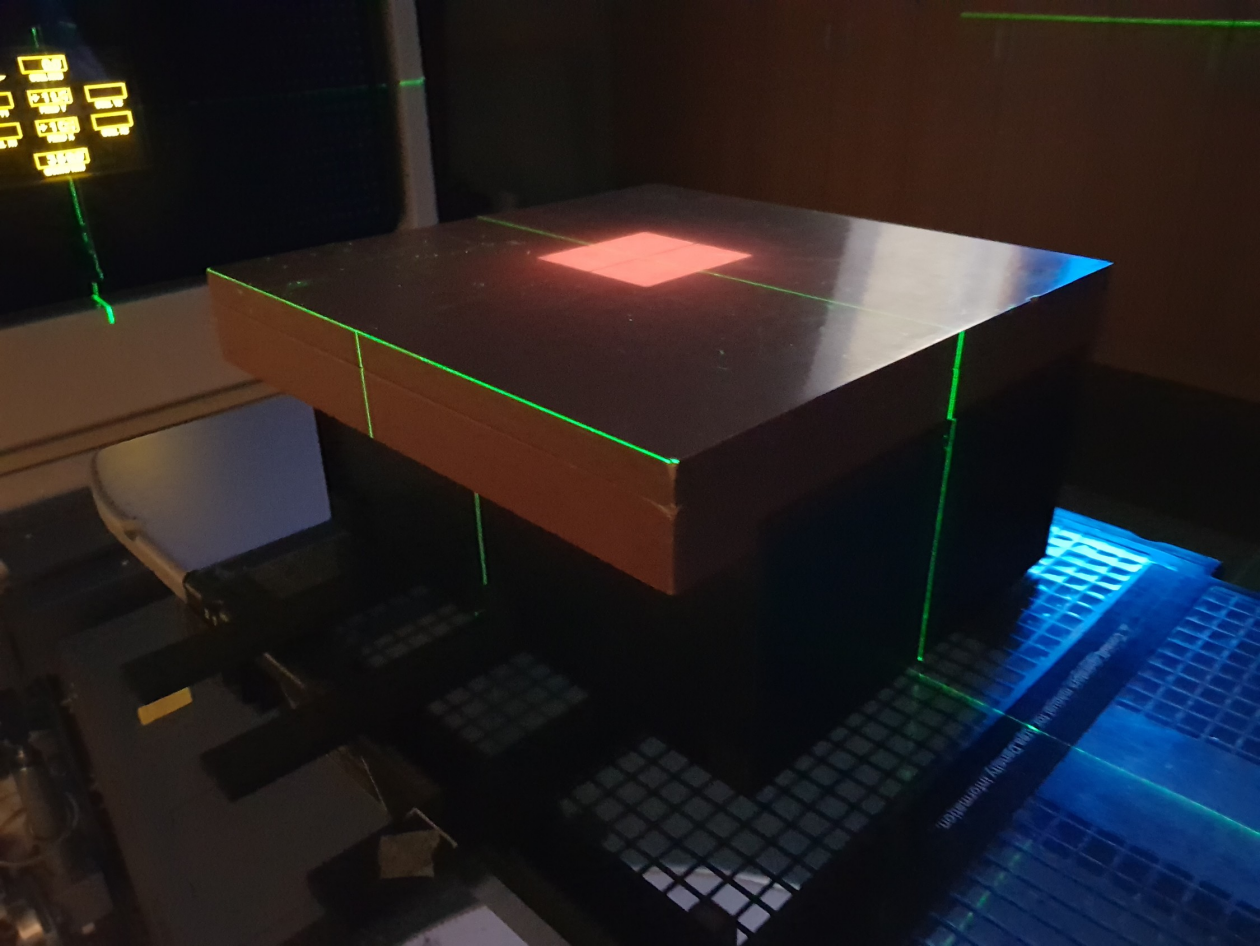
Image of the solid water phantom, 5.0 cm above the prototype of the TRMLSD with a 10.0 × 10.0 cm^2^ field.

The first step was to correct the change in brightness due to the varying distance between the point of the scintillation source and the camera sensor and the inhomogeneity of the scintillator components at a depth of 5.0 cm (Fig 6). The next step was to test the variation in brightness according to the size of the radiation fields at a 10.0 cm depth in the phantom. The experiments for verification of relative brightness variation were performed in the 100.0 cm SSD setup.

#### Dependence of brightness on scintillation-source-point-to-lens distance

**Fig 7:**
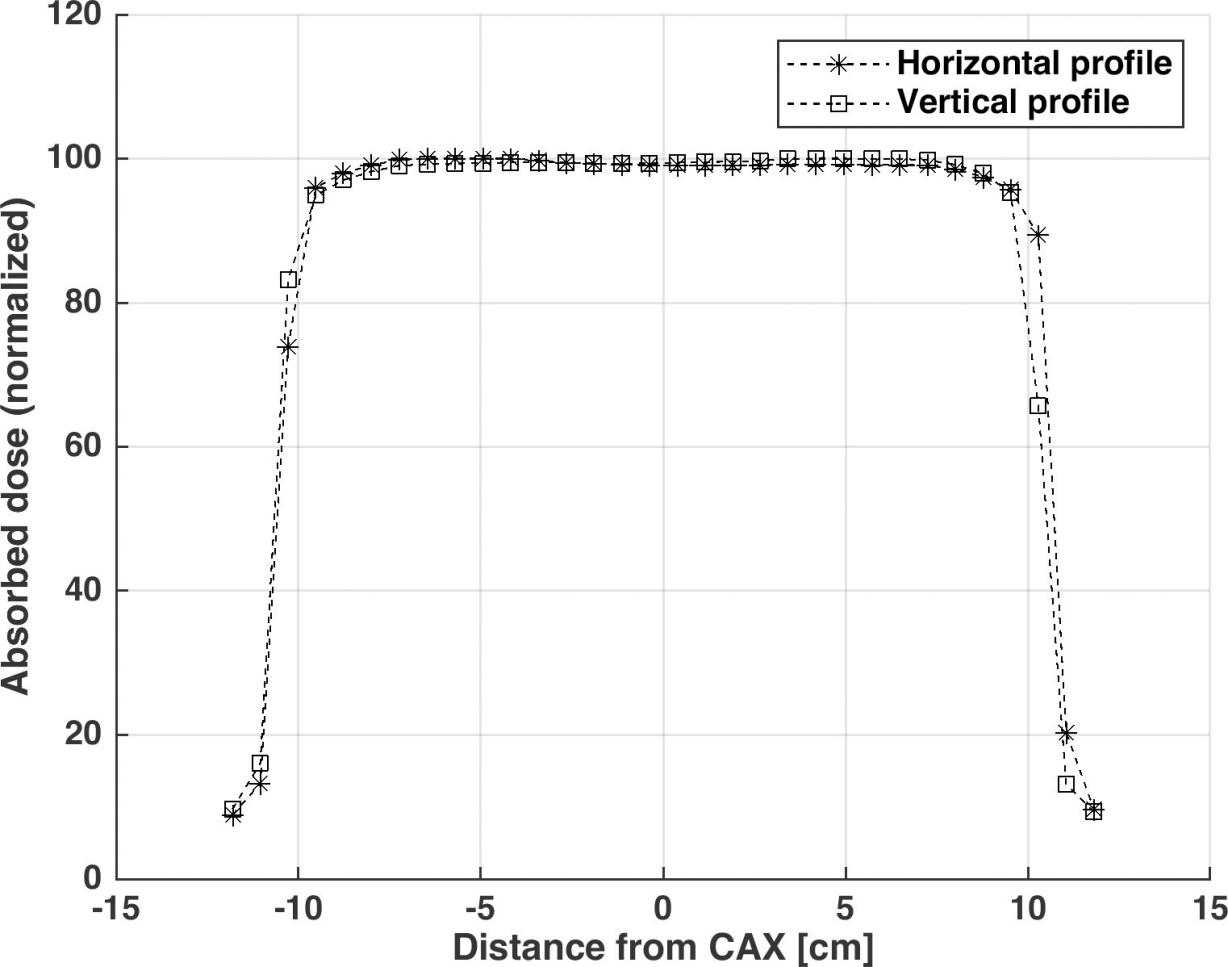
20.0 × 20.0 cm^2^ open field measured by an ion chamber array at the depth of 5.0 cm in the water phantom with an SSD of 100.0 cm.

The flux of light generated from a point source decreases based on the inverse square of the distance. Because the camera is installed at a tilted angle, the distance between each point on the scintillation plate and the pixels of the image sensor varies. Consequently, the brightness of the captured image decreases with respect to the inverse square of the distance. The brightness variation due to both the distance and the chemical inhomogeneity of the scintillator was fixed by obtaining a 2D correction map using a 20.0 × 20.0 cm^2^ open field at a depth of 5.0 cm in the water phantom. The horizontal and vertical profiles of the 20.0 × 20.0 cm^2^ open field shown in Fig 7 were measured using a well-calibrated ion chamber array detector (MatriXX, Ion Beam Application, Belgium). The mean flatness and symmetry of the 20.0 × 20.0 cm^2^ open field were 1.76%, and 0.70%, respectively, and were calculated according to TG-45 protocol. The SSD was set to 100.0 cm, and the dose rate was fixed at 400 MU/s.

#### Dependence of brightness on the radiation field size

The relative output of the linac varied with the radiation field size because of the change in the interaction of the scattered photons with the medium. The brightness measured by the TRMLSD also tended to increase with increasing radiation field size. However, the output factor of the TRMLSD was different from that measured by the ionization chamber and varied with the ISO value. Thus, the variation in brightness was tested for four different field sizes in the range of 2.0 × 2.0 cm^2^ to 20.0 × 20.0 cm^2^.

The dependence of the scintillation intensity on the radiation field size was tested at a depth of 10.0 cm in a water-equivalent solid phantom positioned at 100.0 cm from the source (SSD 100.0 cm). The output factors for different field sizes were measured separately with a 0.13 cm^3^ ionization chamber (CC13; Scanditronix Wellhofer, TN, USA) at a depth of 10.0 cm in a water phantom with full backscatter.

### Deconvolution for penumbra correction with a CNN

When the 2D dose distribution was measured using the TRMLSD with an ISO value of 800 [M (x, y)], the penumbra regions were inaccurately measured owing to the ISO setting (Fig 2C). Moreover, M (x, y) includes complex errors such as those arising from the lack of backscatter, flatness of the 2D correction map, and optical blurring. To accurately measure 2D dose distributions with the TRMLSD, a 2D dose distribution deconvolution is necessary to recover the true dose distribution [D (x, y)] from M (x, y). We hypothesize that the complex errors of M (x, y) could be simultaneously corrected using a CNN consisting of multiple convolution layers, biases, and non-linear activation function.

D (x, y) was calculated using a TPS with the following geometric conditions, which are used for routine patient-specific quality assurances for intensity modulated radiation therapy in our clinic: 5.0 cm water depth, SSD of 95.0 cm, and zero-degree gantry angle. In this study, D (x, y) is the ground truth dose distribution for correcting the measurements M (x, y) obtained under the same geometry condition.

A CNN model, called PenumbraNet [34], was designed to recover the dose in the penumbra region of M (x, y). The input was M (x, y), and the output was the corrected 2D dose distribution [C (x, y)]. To train PenumbraNet, pairs of M (x, y) and D (x, y) corresponding to a 5.0 × 5.0 cm^2^ open field and static IMRT plans configured with 348 subfields of multiple sites (H&N, lung, liver) were prepared as training data.

PenumbraNet comprises an input layer, a hidden layer, and an output layer. M (x, y) enters through the input layer and passes through the hidden layer to reach the output layer. The hidden layer is a combination of convolution filters and biases and is initialized to a random number in the range of 0.000–0.001. In the training process, the hidden layer was optimized to minimize the root-mean-squared error between C (x, y) and D (x, y). The total number of training epochs was 60000, and adaptive moment estimation [35] (Adam) was used as an optimizer.

After the optimization process was completed, the optimized parameters of the hidden layer were saved so that they could be independently applied to the test data for TRMLSD performance validation. The test data comprised a 10.0 × 10.0 cm^2^ open field and ten clinical IMRT cases configured with step-and-shoot methods, which were different from the IMRT plans used to train PenumbraNet.

The details of PenumbraNet were described in a previous report [34].

### Validation of TRMLSD performance

The performance of the TRMLSD was validated for five different items with 6 MV X-rays from the Novalis Tx. The output of the Novalis Tx was maintained at ± 0.7% of daily quality assurance (QA). To validate the TRMLSD, 6 MV X-rays at a dose rate of 400 MU/s and a gantry angle of 0° were used.

Verification was performed for four basic characteristics of the open radiation field: (1) dose linearity, (2) dose reproducibility, (3) dose rate dependency, (4) percent depth dose (PDD), and (5) beam profile. In addition, the accuracy was verified for (6) two simple intensity-modulated fields, and (7) 10 clinical static IMRT cases (step and shoot).

**Fig 8:**
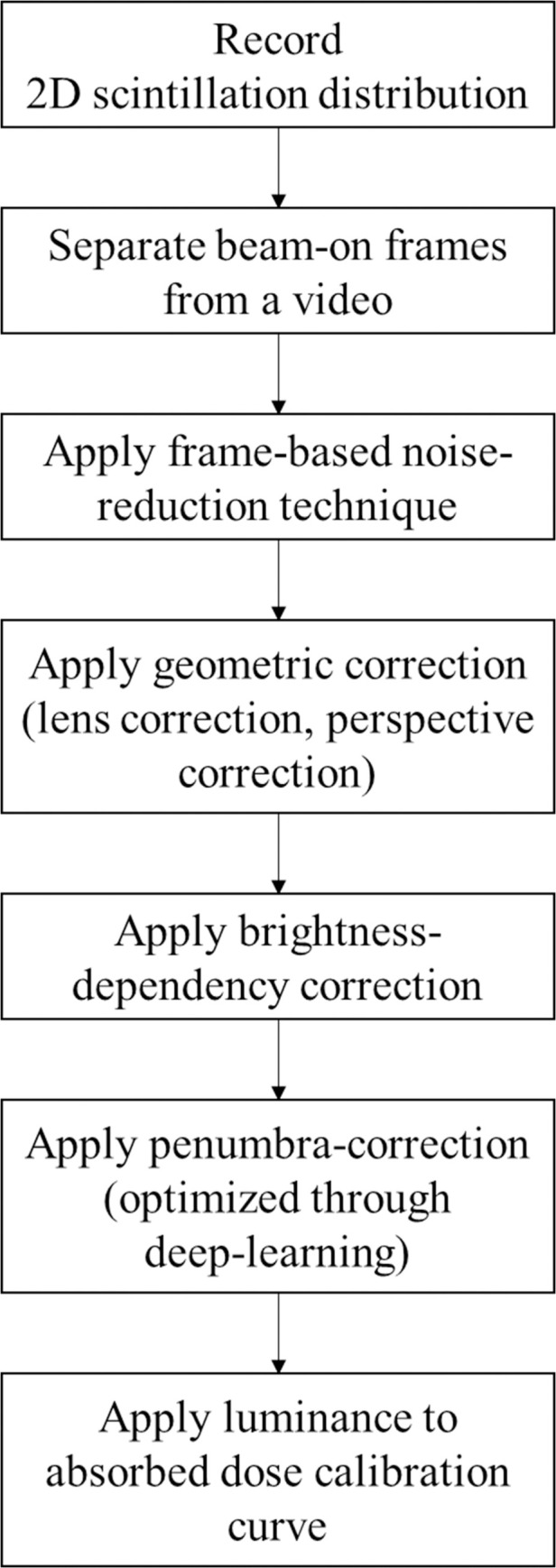
Flowchart for measuring 2D dose distribution using the TRMLSD.

All measurements were made with the TRMLSD and converted to a 2D dose distribution using the algorithms developed in-house (Fig 8). In the case of the EBT3 film analysis, background subtraction was performed for accurate dose analysis, and digitization was performed with the 72-dots-per-inch scanning option using a 11000XL EPSON flatbed scanner. The other 2D dose distributions of the clinical case were calculated using Pinnacle^TM^ (Philips Medical Systems, CA, USA). Analyses were conducted using RIT (RIT Colorado Springs, CO, USA, v6.2) software, and gamma analysis was performed for comparison with the EBT3 film data or D (x, y). Gamma criteria with a 2–4% dose difference and a 2–4 mm distance to an agreement were used. A 5% dose threshold was used.

#### Dose linearity, reproducibility, and dose rate dependency

For the tests, the SSD was 100.0 cm and the dose rate was fixed at 400 MU/s. The geometry conditions of the TRMLSD and ion chamber were those of Setup-A and Setup-B, respectively. A 6 MV photon beam with a 5.0 × 5.0 cm^2^ radiation field was independently irradiated in a range varying from 25 (20.0 cGy) to 249 MU (200.0 cGy) for the TRMLSD and ion chamber. Linearity test data were used as the brightness versus an absorbed dose calibration curve of the TRMLSD. The reproducibility was verified by irradiating the TRMLSD and the ion chamber five times in each geometric condition with a 6 MV photon beam with 25 MU and 249 MU irradiations. In the case of the dose rate dependency test, irradiation at 249 MU was measured three times with the TRMLSD for different dose rates of 100, 300, and 400 MU/min.

Geometric correction, brightness variation correction, noise reduction, and penumbra correction were sequentially applied to analyze the 2D dose distribution. Finally, the brightness data were converted to the absorbed dose by applying a calibration curve.

#### Percent depth dose

A 6 MV photon beam with a 10.0 × 10.0 cm^2^ field and gantry angle of 0° was applied to verify the capability of the TRMLSD for dosimetry of the absorbed dose at varying depths. The SSD and dose rate were fixed at 100.0 cm and 400 MU/s, respectively. The PDD was measured at depths of 2.0, 5.0, 10.0, and 15.0 cm in the solid water phantoms irradiated with 249 MU at each depth, and the PDD was compared with the measurements from the ionization chamber. The data measured with the TRMLSD and ionization chamber were normalized to the dose at 5.0 cm for comparison.

#### Profile verification

The profile of a 10.0 × 10.0 cm^2^ open field was measured using the TRMLSD and EBT3 film (Energy: 6 MV, SSD: 95.0 cm, 5.0 cm thick solid water phantom, 249 MU). In addition, the profile measured by the ionization chamber (CC13) was compared. The EBT3 films measured in this study were scanned using EPSON 11000XL. The flatness, symmetry, and penumbra were analyzed for two datasets using the RIT software and were compared according to the AAPM TG-45 [36].

#### Two simple intensity-modulated plans

**Fig 9:**
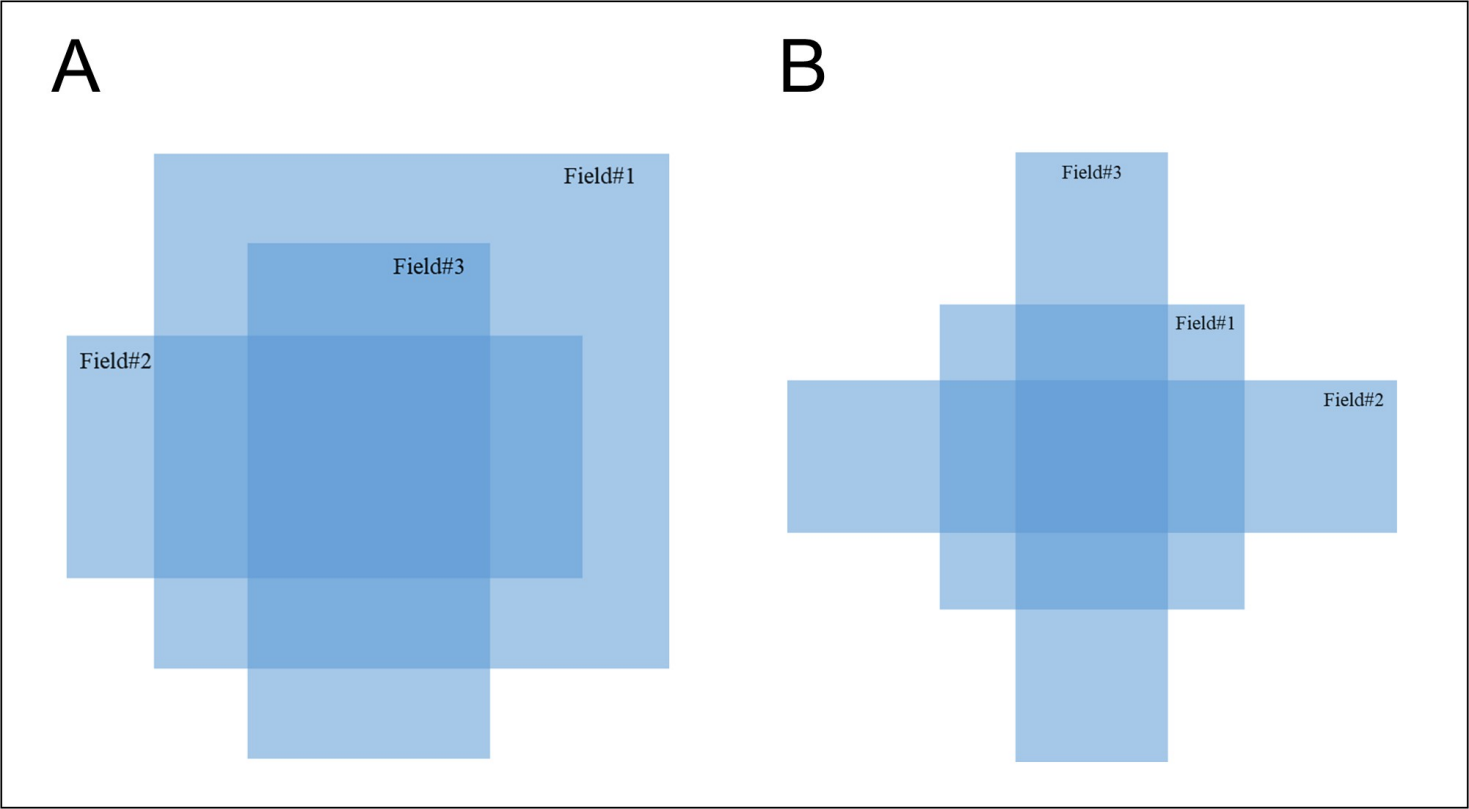
Simple intensity-modulated plans: (A) Field type 1 and (B) Field type 2.

To evaluate the capability of the TRMLSD for simple intensity-modulated plan dosimetry, two different simple intensity modulation plans entailing different sizes and doses of rectangular fields smaller than 10 × 10 cm^2^ were used (Fig 9). The preset MUs for the three fields were 249, 50, and 25 MU, respectively. The simple IMRT plans were delivered to a 30.0 × 30.0 × 5.0 cm^3^ solid water phantom. The SSD and dose rate were set to 95.0 cm and 400 MU/s, respectively. TRMLSD data were analyzed using an in-house program at a time resolution of 1/24 s. Finally, the time-resolved 2D dose distributions were obtained and compared with the data obtained from the EBT3 film. The gamma passing rate was calculated from the 2%/2 mm to 4%/4 mm criteria. The dose threshold was 5% of the maximum dose for each field.

#### Clinical IMRT plans

The final validation of the TRMLSD was performed for pQA using the test data obtained from two heads and necks (7 fields, 7 fields), two livers (5 fields, 5 fields), two lungs (6 fields, 5 fields), two brains (5 fields, 7 fields), one pelvis (7 fields), and one prostate (7 fields). The scintillation sheets of the TRMLSD and EBT3 film were placed under a 5.0-cm-thick solid water phantom at a 100.0 cm SAD. The gantry was fixed at zero degrees. After the measurements, the per-field TRMLSD measurements were compared with D (x, y) using the gamma analysis method with 2%/2 mm and 3%/3 mm gamma criteria. For the prostate case, the accumulated dose distribution measured by TRMLSD was compared with the results of the EBT3 film using gamma analysis with gamma criteria ranging from 2%/2 mm to 4%/4 mm. The dose threshold was 5% of the maximum dose for each field.

## Results

### Brightness dependence correction: 2D correction map

The 2D dose distribution was measured accurately by correcting the brightness dependence due to distance and the chemical inhomogeneity of the scintillation with a 20.0 × 20.0 cm^2^ open field.

**Fig 10:**
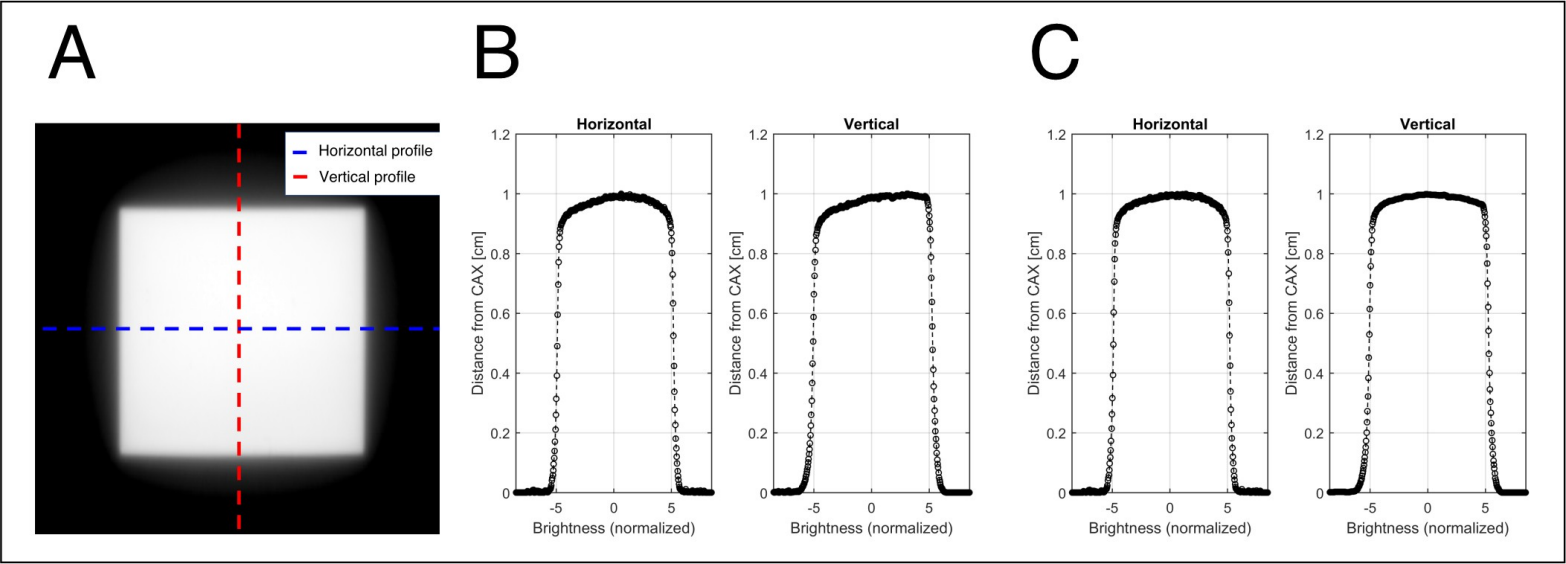
Procedure for brightness dependency by distance: (A) Cumulative two-dimensional scintillation distribution, (B) profiles before the light-intensity correction, and (C) profiles after the light-intensity correction.

Each pixel value in the images captured by the TRMLSD was normalized to the pixel value of the radiation isocenter, and then, the inverse of each pixel value was saved as a 2D correction map. The correction map was applied by element-wise multiplications of the scintillation distribution images obtained with 249 MU irradiation of a 6 MV X-ray beam (10.0 × 10.0 cm^2^ field, 5.0-cm solid water phantom on the TRMLSD). The result of the brightness dependence correction is shown in Fig 10.

### Brightness dependence correction: ISO value

The ratios of brightness were analyzed for different ISO values (Table 1) and compared with the output factor normalized to a 10.0 × 10.0 cm^2^ field. When the ISO value was set to 800, the dose in the penumbra region was lost; nevertheless, the ISO value was still set to 800 considering the output factor.

**Table 1:** Output factor of an ionization chamber and TRMLSD for two ISO values.

	Square field size [cm^2^]			
	2	5	10	20
Ionization chamber (CC13) [1]	0.782	0.892	1.000	1.104
TRMLSD (ISO: 1600) [2]	0.824	0.905	1.000	1.098
TRMLSD (ISO: 800) [3]	0.792	0.896	1.000	1.116
Difference (%): ([2]–[1])*100	4.119	1.292	0.000	–0.663
Difference (%): ([3]–[1])*100	0.978	0.400	0.000	1.122

* All data normalized to the absorbed dose on a 10.0 × 10.0 cm^2^ flat field.

### Penumbra correction using PenumbraNet

The performance of PenumbraNet for accurate dose measurement in the penumbra region using the TRMLSD was validated by loading the parameters of the optimized PenumbraNet and independently applying them to the test data.

**Fig 11:**
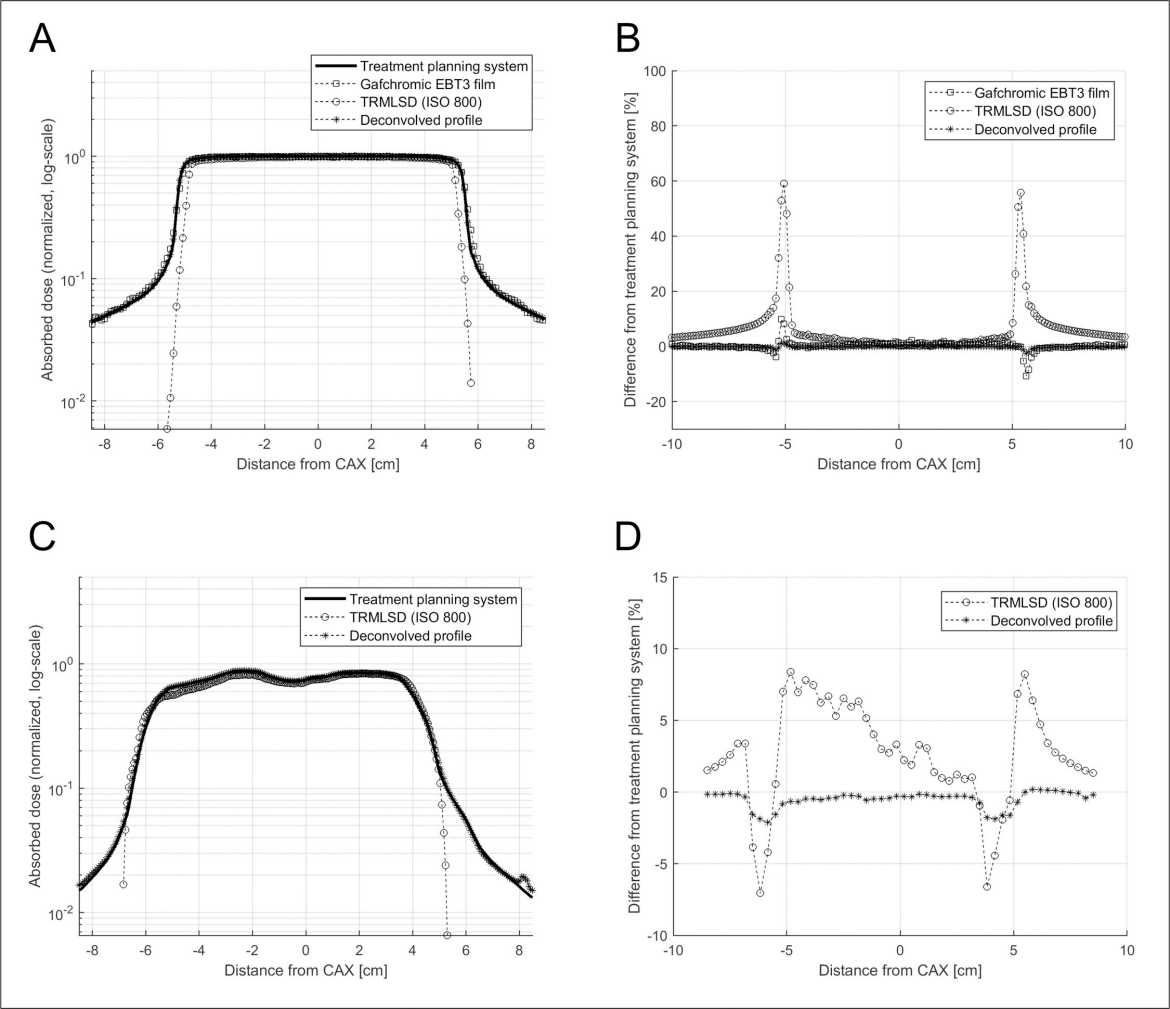
(A) Profiles of the 2D dose distribution for 10.0 × 10.0 cm^2^ open field, (B) Dose differences of the measurements of the 10.0 × 10.0 cm^2^ field with the corrected dose D (x, y), (C) Profiles of the 2D dose distribution for the IMRT plan. (D) Dose differences of measurements of IMRT plan and corrected dose D (x, y): calculated dose distribution corresponding to the TPS (denoted by the solid line), measured dose distribution corresponding to the EBT3 film (denoted by the squares), TRMLSD (denoted by the circles), and correction using PenumbraNet (deconvolved profile, denoted by the stars).

Fig 11A depicts the profiles of M (x, y) given by the TRMLSD and Gafchromic EBT3 film, C (x, y), and D (x, y) for the middle of the 10.0 × 10.0 cm^2^ field. The horizontal dose difference from C (x, y) is shown in Fig 11B, while the profiles of M (x, y), C (x, y), and D (x, y) of the field of the static IMRT plan for pelvis cancer are shown in Fig 11C. Finally, Fig 11D shows the horizontal dose difference from C (x, y). The details of PenumbraNet are reported elsewhere [34].

### Dose linearity, reproducibility, and dose rate dependency

**Fig 12:**
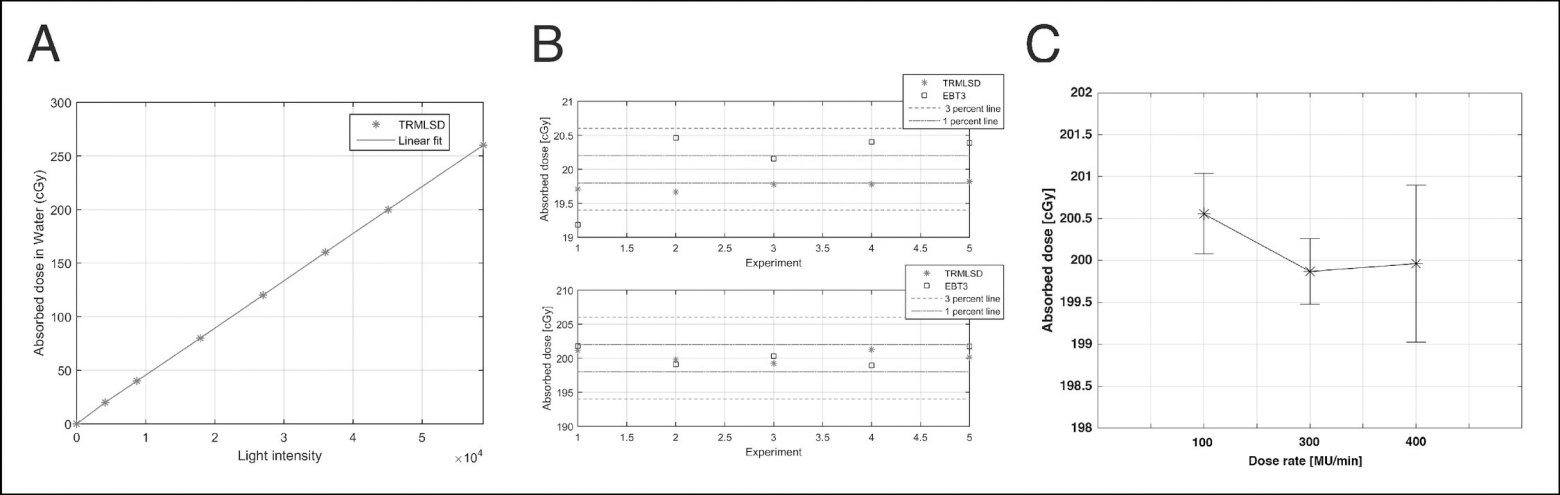
Test results for (A) does linearity, (B) reproducibility, and (C) dose rate dependency of TRMLSD.

Dose linearity, reproducibility, and dose rate dependency are important characteristics of a dosimeter. The response of the developed TRMLSD to the absorbed dose was tested by analyzing the linear relationship between the brightness and the absorbed dose with 6 MV photon beams. The R-squared value of the linear fitting of the measurement data was 0.9998, and the root-mean-square error was 1.010 (Fig 12A). The brightness measured by the TRMLSD was converted to the absorbed dose using this curve.

The reproducibility of the TRMLSD was tested for 20.0 cGy and 200.0 cGy. The mean and standard deviations of each set of five measurements were 19.99 ± 0.15 cGy and 202.69 ± 0.82 cGy for 20.0 cGy and 200.0 cGy irradiation, respectively. The mean and standard deviation of each set of five measurements using the EBT3 film were 20.12 ± 0.54 cGy and 200.37 ± 1.36 cGy, respectively (Fig 12B). The dose rate dependency was tested for 100, 300, and 400 MU/min by delivering 200 cGy. The mean and standard deviation of each dose rate for three measurements were 200.55 ± 0.48 cGy, 199.87 ± 0.39 cGy, 199.96 ± 0.93 cGy, respectively (Fig 12C).

### Percent depth dose

**Fig 13:**
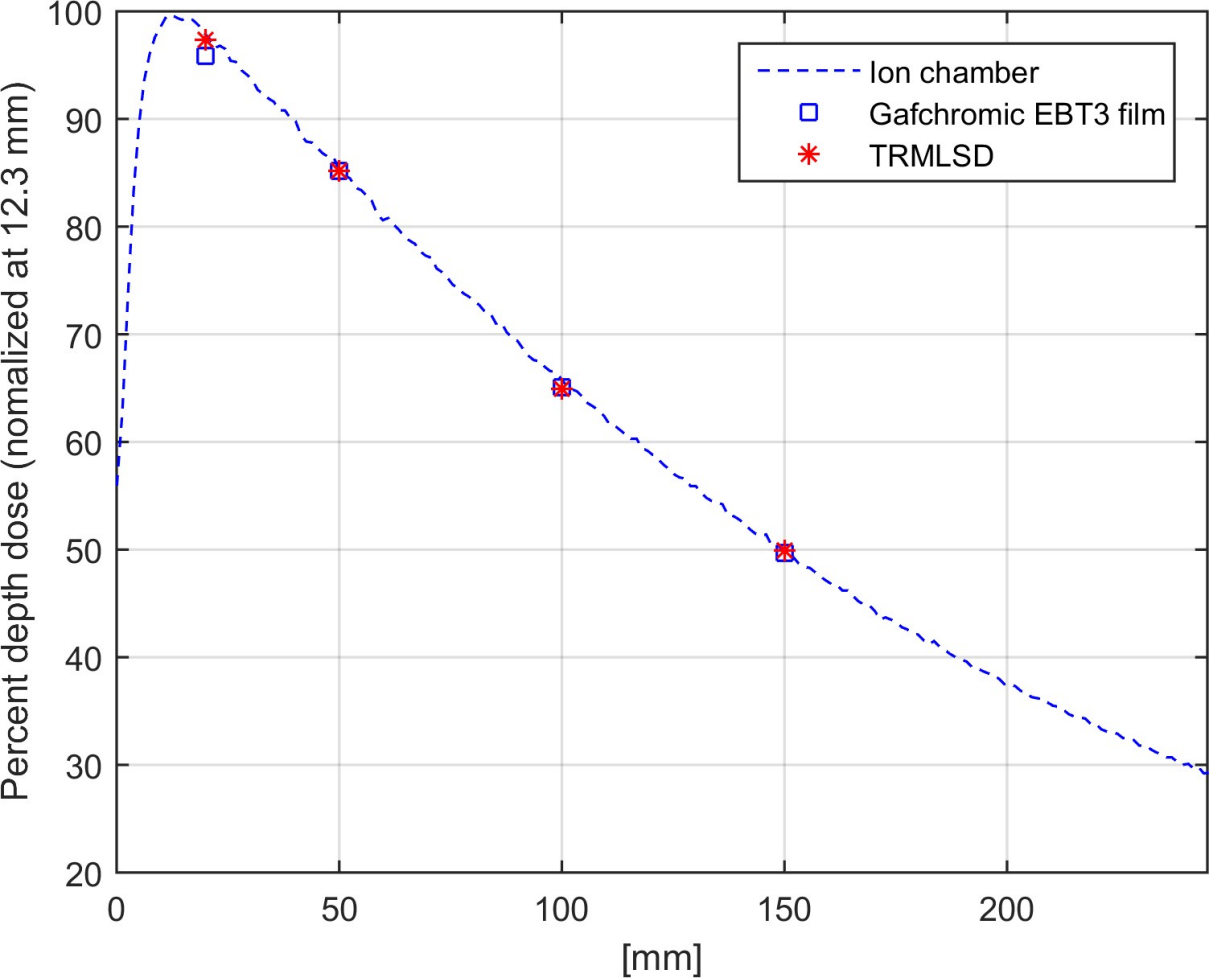
PDD profile measured by the TRMLSD (denoted by stars), EBT3 film (denoted by squares), and ionization chamber (denoted by a dashed line) for a 6 MV X-ray. TRMLSD and EBT3 film data were normalized to the ionization chamber data at a depth of 5.0 cm.

The measurement data for the ionization chamber were normalized to the dose at the maximum dose depth (12.3 mm). The TRMLSD measurements were matched to the percentage at 5.0 cm of the ionization chamber data (85.2%) and are plotted in Fig 13. The relative errors with respect to the ionization chamber data were −1.51%, 0.19%, and −0.23% with the TRMLSD, and −2.16%, −0.80%, and −1.99% with the EBT3 film at 2.0, 10.0, and 20.0 cm, respectively. The mean of the relative errors between the TRMLSD and EBT3 films was less than 0.64% and 1.65%, respectively.

### Beam profile verification

The flatness, symmetry, and penumbra size are presented in Table 2. The measured flatness in the cases of the EBT3 film and TRMLSD differed from those corresponding to the ionization chamber by −0.16% and 0.1%, respectively. For symmetry, the difference was 0.2% and 0.3%, respectively, and for penumbra size, the difference was less than 0.5 mm for both cases.

**Table 2:** Beam profiles of 6 MV X-rays for a 5.0-cm-thick solid water phantom.

Measurement tool	Flatness (%)	Symmetry (%)	Penumbra (cm)
Ionization chamber	1.78	0.83	L: 0.85
			R: 0.85
EBT3 film	1.62	0.85	L: 0.42
			R: 0.47
TRMLSD	1.88	0.86	L: 0.51
			R: 0.51

### Simple intensity-modulated fields

**Fig 14:**
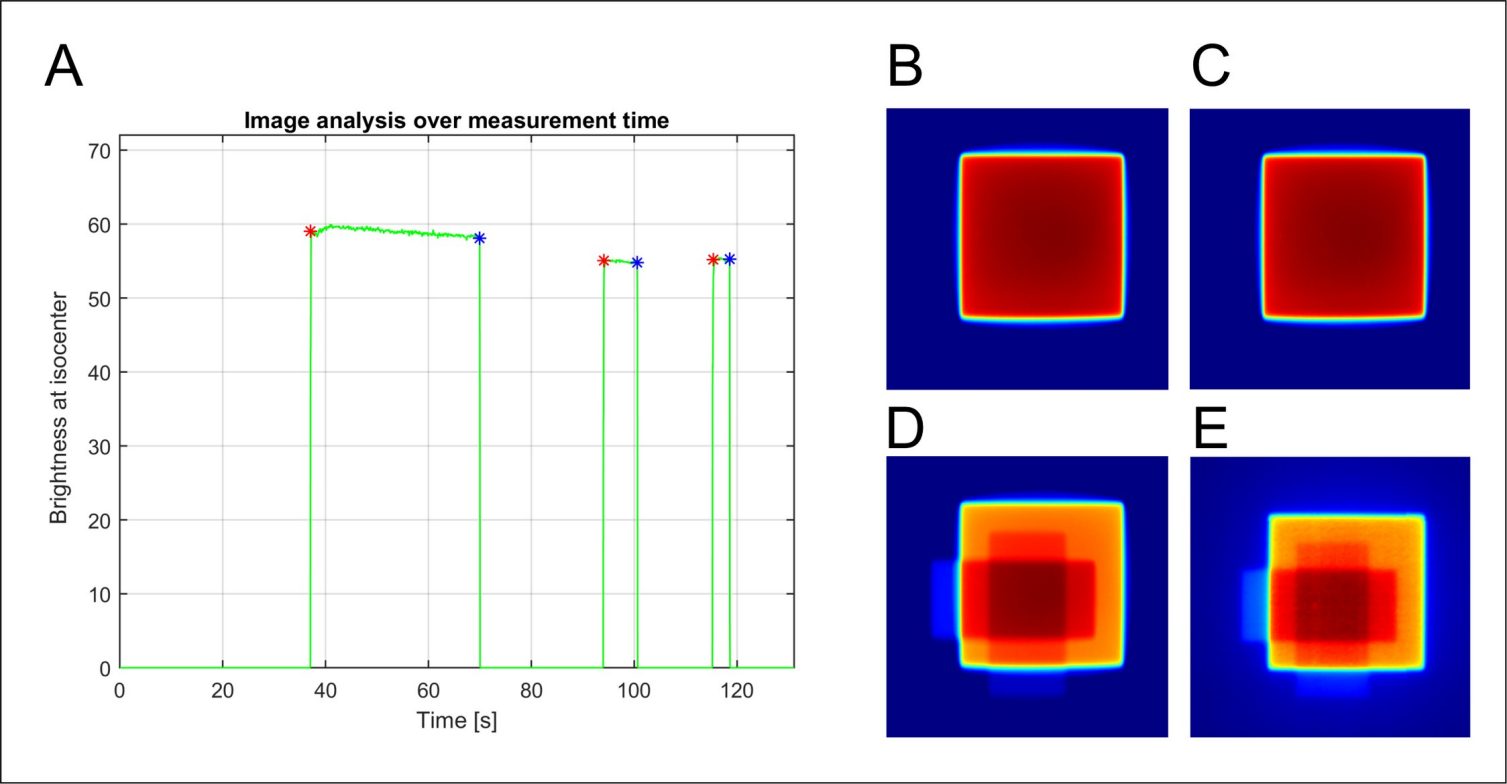
(A) Scintillation signal over time for a simple intensity-modulated plan (field type 1). The accumulated 2D dose distribution of the TRMLSD at (B) 81.6, (C) 110.4, and (D) 127.2 s. (E) 2D dose distribution of the EBT3 film.

To evaluate the possibility of clinical application of the TRMLSD, we tested the accuracy of the method for simple intensity-modulated plans. In the case of field type 1, the total measurement time was 131.1 s, and the irradiation periods of the three fields were 32.67, 6.29, and 3.34 s (Fig 14A). A gamma comparison was performed for the accumulated dose overtime at 81.6, 110.4, and 127.2 s (Fig 14B and 14D). The obtained agreements of the 2D dose distribution were 94.72%, 97.84%, and 96.89% with a 3%/3 mm gamma criterion between the TRMLSD and EBT3 film (Fig 14E). In the case of field type 2, the gamma passing rates for the accumulated 2D dose distribution for each field were 94.63%, 95.85%, and 96.64%. The gamma passing rates under different criteria are summarized in Table 3.

**Table 3:** Gamma analysis between the TRMLSD and EBT3 film for different gamma criteria.

	Field type 1			Field type 2		
	Accumulation time (s)			Accumulation time (s)		
Gamma criterion	81.6	110.4	127.2	81.6	110.4	127.2
2%/2 mm	91.59	93.47	92.93	92.87	92.06	93.75
3%/3 mm	94.72	97.84	96.89	94.63	95.85	96.64
4%/4 mm	99.93	99.99	99.86	99.75	99.99	99.72

### Clinical IMRT cases

**Fig 15:**
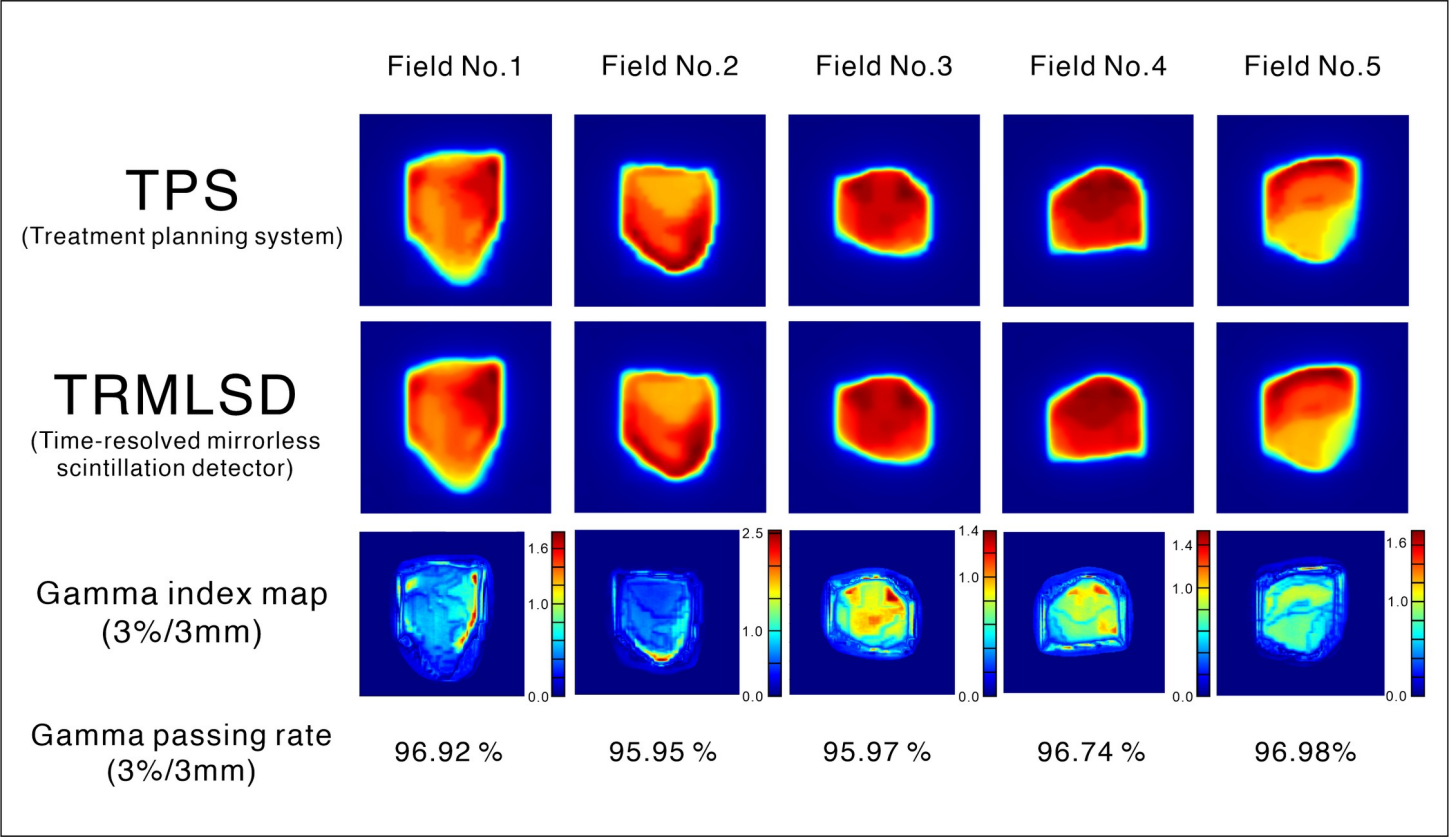
Each column represents a different field in the clinical liver IMRT case. The first row shows the normalized 2D dose distribution calculated by the treatment planning system (TPS). The second row shows the normalized 2D dose distribution acquired using the TRMLSD. The third row shows the gamma index maps with the passing rates for each plan. A 3%/3 mm gamma criteria suppressing 5% of the maximum dose and global normalization were used. The TPS data were used as the reference dataset for comparison.

The pQA for individual fields was performed for the 10 clinical IMRT cases using the TRMLSD. The TRMLSD analyzed the 2D dose distribution over time. The gamma passing rates were calculated per field for the 10 IMRT cases. The mean gamma passing rates of the subfields were 88.96% and 95.75% for the 2%/2 mm and 3%/3 mm gamma criteria, respectively. The quantitative results are summarized in Table 4. For Liver (1), the 2D dose distributions of the TPS, TRMLSD, and gamma index maps with the passing rates of 3%/3 mm are shown in Fig 15.

**Table 4:** Quantitative results of the gamma passing rate per field dose distribution with 2%/2 mm and 3%/3 mm gamma criteria for the 10 IMRT cases (Unit: %).

Gamma	H&N (1)							
criteria	1^st^ field	2^nd^ field	3^rd^ field	4^th^ field	5^th^ field	6^th^ field	7^th^ field	Avg.
3%/3 mm	95.11	93.32	94.83	95.47	95.60	95.28	97.27	95.27
2%/2 mm	87.96	86.93	87.18	89.72	89.27	87.71	90.07	88.41
	H&N (2)							
	1^st^ field	2^nd^ field	3^rd^ field	4^th^ field	5^th^ field	6^th^ field	7^th^ field	Avg.
3%/3 mm	94.02	95.08	94.01	95.17	94.11	94.17	94.79	94.48
2%/2 mm	87.31	88.06	87.38	87.31	88.09	87.43	88.37	87.71
	Liver (1)							
	1^st^ field	2^nd^ field	3^rd^ field	4^th^ field	5^th^ field			Avg.
3%/3 mm	96.92	95.95	95.97	96.74	96.98			96.51
2%/2 mm	90.57	89.52	88.88	91.23	90.79			90.20
	Liver (2)							
	1^st^ field	2^nd^ field	3^rd^ field	4^th^ field	5^th^ field			Avg.
3%/3 mm	98.03	97.62	95.02	98.18	98.25			97.42
2%/2 mm	91.36	90.52	88.79	90.1	91.98			90.55
	Lung (1)							
	1^st^ field	2^nd^ field	3^rd^ field	4^th^ field	5^th^ field	6^th^ field		Avg.
3%/3 mm	96.80	93.84	94.68	93.38	96.24	96.72		95.27
2%/2 mm	90.31	88.54	88.68	88.23	88.89	88.21		88.81
	Lung (2)							
	1^st^ field	2^nd^ field	3^rd^ field	4^th^ field	5^th^ field			Avg.
3%/3 mm	96.69	96.54	95.51	96.70	96.71			96.43
2%/2 mm	91.318	89.378	89.818	90.408	90.928			90.37
	Brain (1)							
	1^st^ field	2^nd^ field	3^rd^ field	4^th^ field	5^th^ field			Avg.
3%/3 mm	95.08	96.14	94.07	96.23	95.17			95.33
2%/2 mm	89.66	89.41	86.73	89.66	89.44			88.98
	Brain (2)							
	1^st^ field	2^nd^ field	3^rd^ field	4^th^ field	5^th^ field	6^th^ field	7^th^ field	Avg.
3%/3 mm	96.07	93.30	94.00	94.53	94.04	95.04	95.56	94.65
2%/2 mm	88.67	86.39	86.97	88.12	86.77	86.63	88.22	87.39
	Pelvis							
	1^st^ field	2^nd^ field	3^rd^ field	4^th^ field	5^th^ field	6^th^ field	7^th^ field	Avg.
3%/3 mm	95.21	96.06	95.04	95.91	96.12	94.09	96.11	95.67
2%/2 mm	88.58	88.33	87.03	86.97	88.64	86.42	89.35	87.91
	Prostate							
	1^st^ field	2^nd^ field	3^rd^ field	4^th^ field	5^th^ field	6^th^ field	7^th^ field	Avg.
3%/3 mm	96.89	95.96	97.35	97.69	98.68	96.99	98.05	97.37
2%/2 mm	87.36	91.64	92.22	91.45	92.00	92.51	86.43	90.52

For the 2D dose distribution of the liver plan, the results obtained using the TRMLSD and EBT3 film were compared with the 2D dose distribution of the TPS using gamma analysis with criteria from 2%/2 mm to 4%/4 mm. The pass rates with the 3%/3 mm criterion were 99.00% and 95.51% for the TRMLSD and EBT3 films, respectively. The gamma passing rates with various criteria for the accumulated fields are summarized in Table 5.

**Table 5:** Gamma passing rate of accumulated dose with the TRMLSD and EBT3 film compared with the TPS.

	Gamma criteria		
	2%/2 mm	3%/3 mm	4%/4 mm
TRMLSD	96.98	99.00	99.71
EBT3 film	93.32	95.51	96.74

## Discussion

In this study, a compact scintillation detector system without a mirror (TRMLSD) was proposed to perform high-resolution 2D dose distribution measurements using a camera with a CMOS image sensor and a sheet-type inorganic scintillator.

The major issues faced by the TRMLSD during the development process are (1) a dosimetric error in the penumbra region due to the ISO value parameter of the digital camera and (2) backscatter due to the structural characteristics of the scintillation detector.

The ISO value of a digital camera controls the light sensitivity of the digital imaging sensor. Particularly, this value directly affects the amplifier transistor located before the analog-to-digital converter. Thus, different ISO values cause a significant difference in the measured profile, specifically in the low-dose region, which corresponds to low light signals. In particular, uncertainty in the penumbra region could introduce a significant dosimetric error; this is because the intended dose distribution of an IMRT plan is achieved by irradiating many subfields such that the outfield dose distribution of each subfield actually contributes to the infield dose distribution. Moreover, for a structural reason, the effect of backscatter on M (x, y) was not considered with regard to collecting the scintillation using the camera. Thus, the deep-learning model (PenumbraNet) was developed to simultaneously solve these issues, and its efficacy and accuracy were verified.

The cornerstones of this study are as follows: (1) We removed the mirror from the design of the conventional scintillation detector; thus, the volume and weight of the dosimeter were dramatically reduced. In our case, 16.52 kg was reduced. (2) The 2D dose distributions perpendicular to the beam axis were measured with clinically acceptable accuracy for patient-specific IMRT QA configured with a step-and-shoot method owing to the image processing techniques and convolutional neural network. (3) The scintillation collection device used in this study (HERO5 Black camera) was lightweight and wireless; thus, the TRMLSD was a wireless detector. (4) The designed scintillation detector can perform time-resolved 2D dosimetry through frame-based analysis of sequentially recorded scintillation images (video), and thus, can analyze the 2D dose distribution per segment and/or field.

However, the following factors may affect the measurement accuracy: (1) The quenching effect has been reported to occur during particle dosimetry, but it is negligible (less than 1%) in photon therapy [37]. (2) A spatial error can occur in the geometric correction process when obtaining 2D planar dose distributions without a mirror. Thus, the accuracy of the geometric calibration was validated using a test plan designed to verify the rotational invariance of the calibration, with dose distributions corresponding to three stripes of different widths (10.0 × 1.0 cm^2^, 10.0 × 2.0 cm^2^, and 10.0 × 4.0 cm^2^). Two measurements were performed under different conditions to verify the rotational invariance of calibration: one measurement was conducted along the axial direction, and the other by rotating the TRMLSD at an angle of 90°. No directional dependency was proven because the mean gamma passing rate of the two different binary maps with a 1%/1 mm gamma criterion, based on 50% of the maximum value, was 99.85% (0 < 50% and 1 ≧ 50%). The binary map of the irradiated field corresponds to the field shapes. (3) The method for correcting the brightness variation in 2D, which is generated using a 20 × 20 cm^2^ homogeneous radiation field, could cause an error in dosimetry because of the non-perfect flatness of the radiation field. There is less than about a 1.0% difference in the effective measurement area (17.6 × 17.6 cm^2^). If a homogeneous light source such as an LCD is used instead of a scintillator sheet, the variations in distance can be compensated for; however, the inhomogeneity of the scintillator sheet cannot be considered. (4) The FBNR technique has the potential to cause a dosimetric error for continuously varying dose distributions. This is because the hot pixel was discriminated using the instantaneous characteristics of the hot pixel. For dosimetry with continuously varying dose distributions such as in dynamic IMRT using the TRMLSD, a median filter [38] and the dark-frame technique [39] can potentially improve hot pixel reduction. (5) The effect of the SSD on the backscatter factor, defined as the ratio of the absorbed dose for a particular backscatter thickness to that in the case of full backscatter under a particular square field size and depth, was not considered in this study. If the TRMLSD is used in a fixed position (i.e., SAD is 100.0 cm), errors can be reduced by employing the aforementioned correction techniques for that specific position.

In summary, the basic characteristics of the TRMLSD, including linearity, reproducibility, and dose rate dependency, were verified, and its usability in measuring the PDD for simple intensity-modulated cases was shown to agree well with those of the ionization chamber and EBT3 film. Its capability for clinical step-and-shoot IMRT dosimetry was also verified, proving its good agreement with a TPS. The TRMLSD, which utilizes an action camera that has not been considered for dosimetry before, can not only provide mobility but can also achieve acceptable accuracy in 2D dosimetry with the incorporation of software corrections. Moreover, the TRMLSD can be used as a portable and independent dose-measurement device for QA in IMRT.

## Conclusions

In this work, we studied the feasibility of using the TRMLSD for the measurement of the 2D dose distribution of a photon beam. To overcome the inherent shortcomings of the design, we have successfully developed and applied techniques, including computer vision techniques and the deep-learning model based on a CNN, for geometric correction, brightness variation correction, FBNR, and backscatter and penumbra correction. The TRMLSD exhibited good performance for clinical IMRT plans configured with the step-and-shoot method. The verified accuracy and time-resolved characteristics of the dosimeter may be useful not only for the quality assurance of machines but also for patient-specific quality assurance for clinical IMRT plans.

The TRMLSD can be a dynamic dosimetry tool for clinical IMRT plans for dynamic therapies, such as IMRTs and volumetric arc therapy with X-rays, and scanning methods for proton and carbon therapies in which the beam fluence and characteristics change over time.
